# Color demosaicking via fully directional estimation

**DOI:** 10.1186/s40064-016-3380-1

**Published:** 2016-10-06

**Authors:** Lingyan Fan, Guorui Feng, Yanli Ren, Jinwei Wang

**Affiliations:** 1 Micro-Electronics Research Institute, Hangzhou Dianzi University, Zhejiang, China; 2 School of Communication and Information Engineering, Shanghai University, Shanghai, China; 3 Nanjing University of Information Science and Technology, Jiangsu, China

**Keywords:** Color filter array, Demosaicking, Multiscale color difference, Fully directional estimation

## Abstract

Given a natural image from the single sensor, the key task is to properly reconstruct the full color image. This paper presents an effectively demosaicking algorithm based on fully directional estimation using Bayer color filter array pattern. The proposed method smoothly keeps access to current reconstruction implementations, and outperforms the horizontal and vertical estimating approaches in terms of the perceptual quality. To analyze the target of existing methods, the proposed algorithm use the multiscale gradients in single green channels as the diagonal information for the auxiliary interpolation. Furthermore, two group of weights (one is from the horizontal and vertical directions, another is from the diagonal and anti-diagonal directions) are built. Combinational weight is better suited for representing neighbor information. Another contribution is to better use the prior result. While calculating the same type of color difference, we divide all the color difference values into two interleaved parts. Estimated value in the first part will guide the subsequent color difference in the second part. It less brings the artifact of the interpolation procedure. Experimental results show that this adaptive algorithm is efficient both in the objective and subjective output measures.

## Background

**Fig. 1:**
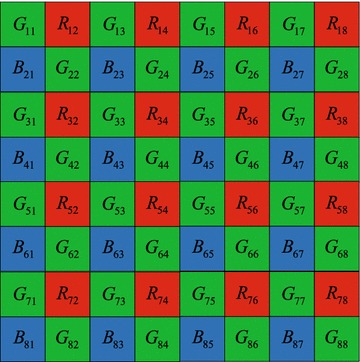
The Bayer patten arrangement

In many consumer electronics systems, such as pocket devices and mobile phones, single imaging sensor devices which are designed based on the color filter array have widely been used for the lower cost. Each pixel in the sensor can only capture the one of color components. The missing color are interpolated by the local or nonlocal similar region. This process is also named color demosaicking. The typical pattern is arranged as Bayer pattern (Bayer 1976) shown in Fig. 1, where the number of the green pixels is twice as the one of red and blue pixels. Because the sensor obtains the true values in the specified color channel, the missed color values have to be reconstructed in the terms of high correlation between all primary color channels. The most common methods in color demosaicking are derived from the color difference correlation property.

High correlation between all pairs of color channels measured over benchmark images indicates a commonly exploited property to devise the interpolation method. As the well-known second-order differential method, Adams and Hamilton (1996) interpolated the missing color values along the smooth edge direction named as ACPI. Motivated by directional interpolation scheme, the latter methods expanded ACPI by enough employing the directional weighted estimator. For example, Zhang proposed the horizontal and vertical direction weights via linear minimum mean square error estimation (Zhang and Wu 2005). In this demosaicking method, the larger directional variance means the smaller weight. Another type of early methods belonged to nonheuristic method. The high frequencies of the green values primarily guided the interpolation of red and blue channels (Gunturk et al. 2002). Later, multiple method fusion was formulated as an optimal problem. By analyzing the color local property, linear minimum mean-square estimation and support vector regression were grouped into a unified scheme (Zhang et al. 2009). Based on the high-frequency information preservation, the effective luminance at three color channels was designed using the Fourier transforms of down-sampled signals (Lian et al. 2007). Two detailed comparisons were also provided for an early assessment of the performance in the famous review papers (Li et al. 2008; Menon and Calvagno 2011).

In previous methods, direction effect is usually calculated by consecutive narrow line edges. Therefore, many instable weights seriously mislead the estimation of color difference. To solve this problem, new edge-sensing measure called integrated gradient can effectively extract gradient variance at the border. The edge indicator could serve as an individual guide to many successive demosaicking methods (Chung and Chan 2010). If we consider all from the digital devices, the prior knowledge from natural images will leverage on the properties of exploring intrinsic correlation (Menon and Calvagno 2009). Recent works showed gradients were the ultimate factor for extracting directional data from digital images (Pekkucuksen and Altunbasak 2013). Multiscale gradients (MG) estimated horizontal and vertical color difference using more scales into the difference equation. If the method properly used the relationships of intra and intercolor correlation and will balance the color difference results (Jaiswal et al. 2014). The interpolation errors based on geometric duality using in the low resolution image partially compensated the missing information in demosaicking (Kim et al. 2014). After the initial interpolation is completed, the refinement method in the distinct channels could significantly improve the ultimate performance (Menon and Calvagno 2011). In fact, the principle of the smoothness of color difference led to the weight fusion in the local image (Zhou et al. 2012). Recently, a type of algorithm based on residual interpolation (RI) was proposed (Kiku et al. 2013). It gives another interpolation structure and uses color residual to interpolate all the missing points. Its succeeding versions (Kiku et al. 2014; Monno et al. 2015; Ye and Ma 2015) generates the distinct definitions of the residuals. RI is greatly efficient to run on the image which has the weaker correlation between colors (Kiku et al. 2016).

The traditional methods in demosaicking depended on the balance between horizontal and vertical directions (Menon and Calvagno 2011; Pekkucuksen and Altunbasak 2013). In this section, we provide a new attempt to estimate the fully directional weight from the color difference and design two group of weights (one is from the horizontal and vertical directions, another is from the diagonal and anti-diagonal directions). It is seldom seen in the aforementioned methods.

## Proposed color demosaicking method

### Green channel estimation

Since the number of the green pixels is the most prevalent, many demosaicking methods try to interpolate all green pixels first. Difference gradient-based interpolation in various directions at each pixel will guide the interpolation along the smooth edge. One solution to tackle the problem of avoiding cross the edge is to adopt the second-order Laplacian interpolation filter (Zhang and Wu 2005). For red and green rows, directional interpolations at red and green points can be given by

1 $$\begin{aligned} R^{-}_{i,j}= & {} \frac{R_{i,j-1}+R_{i,j+1}}{2}+\frac{2G_{i,j}-G_{i,j-2}-G_{i,j+2}}{4} \end{aligned}$$

2 $$\begin{aligned} G^{-}_{i,j}= \frac{G_{i,j-1}+G_{i,j+1}}{2}+\frac{2R_{i,j}-R_{i,j-2}-R_{i,j+2}}{4} \end{aligned}$$

where the superscript − means the operation in the horizontal estimate. Similarly, we compute the vertical estimate as $R^{|}_{i,j}$ and $G^{|}_{i,j}$ at the coordinate (*i*, *j*). The interpolated direction estimate will result in directional color difference shown in

3 $$\begin{aligned} d^{-_{gr}}_{i,j}=\left\{ \begin{array}{ll} G^{-}_{i,j}-R_{i,j},& \text {missing}\quad G_{i,j} \\ G_{i,j}-R^{-}_{i,j},& \text {missing}\quad R_{i,j} \end{array} \right. \end{aligned}$$

and the second order color differential (Pekkucuksen and Altunbasak 2013) followed by

4 $$\begin{aligned} D^{-_{gr}}_{i,j} &= \left| \dfrac{R_{i,j-1}-R_{i,j+1}}{2}-\dfrac{G_{i,j-2}-G_{i,j+2}}{4}\right. \nonumber \\& \quad \left. + \, \dfrac{R_{i,j-3}-R_{i,j+3}}{8}-\dfrac{G_{i,j-4}-G_{i,j+4}}{16}\right| \end{aligned}$$

Multiscale color gradients over a narrow window is equivalent to average the color difference using the lowpass filter. Moreover, we define the second order differential in the main diagonal direction as follows.

5 $$\begin{aligned} D^{\backslash _{gr}}_{i,j} &= \left| \dfrac{G_{i+1,j+1}-G_{i-1,j-1}}{2}-\dfrac{G_{i+2,j+2}-G_{i-2,j-2}}{4}\right. \nonumber \\&\quad \left. + \, \dfrac{G_{i+3,j+3}-G_{i-3,j-3}}{8}-\dfrac{G_{i+4,j+4}-G_{i-4,j-4}}{16}\right| \end{aligned}$$

The second order differential $D^{/_{gr}}_{i,j}$ in the anti-diagonal direction is similarly defined. For the green position, in diagonal directions, only green values can be provided for calculating differential information. Meanwhile, the color differences between green and blue can be obtained in the same way, occurred in the Eqs. (1–5). The green and red pixels combination estimation of the first step are alternatively filtered by

6 $$\begin{aligned} GR_{i,j}=\left(\omega ^{|}{} {\mathbf{f}}\cdot {\mathbf{D}}^{|_{gr}}_{i-2:i+2,j}+\omega ^{-}{\mathbf{D}}^{-_{gr}}_{i,j-2:j+2}\cdot {\mathbf{f}}^T\right)/M_T; \end{aligned}$$

where ${\mathbf{f}}=[1/4, 1/2, 1/4]$ and the operator $\cdot$ denotes the inner product of vectors. ${\mathbf{D}}^{|_{gr}}_{i-2:i+2,j}$ and ${\mathbf{D}}^{-_{gr}}_{i,j-2:j+2}$ are the column and row vectors consisted of $D^{-_{gr}}$ and $D^{|_{gr}}$, respectively. The weights for each direction $(\omega ^{-},\omega ^{|})$ are calculated using color difference gradients in the horizontal and vertical directions as:

$$\begin{aligned} \omega ^{-} &= 1/\left[ \left( \mathop {\sum }\limits _{i-2}^{i+2}\mathop {\sum }\limits _{j-2}^{j+2}D^{-_{gr}}_{i,j}\right) ^4+\varepsilon \right] ,\quad \omega ^{|}=1/\left[ \left( \mathop {\sum }\limits _{i-2}^{i+2}\mathop {\sum }\limits _{j-2}^{j+2}D^{|_{gr}}_{i,j}\right) ^4+\varepsilon \right] ,\\ M_T &= \omega ^{-}+\omega ^{|} \end{aligned}$$

where $\varepsilon$ is a small positive number to avoid zero denominator. $M_T$ normalizes the total weights. Because horizontal and vertical weights simply decompose the edge into two directions. This is not sufficient to represent the edge shape. To better solve this problem, we first detail directional weights as follows

7 $$\begin{aligned} \omega ^{\uparrow } &= 1/\left[ \mathop {\sum }\limits _{k=i-2}^{i}\mathop {\sum }\limits _{l=j-1}^{j+1}\left(D^{|_{gr}}_{k,l}\right)^2+\varepsilon \right] \nonumber \\ \omega ^{\downarrow } &= 1/\left[ \mathop {\sum }\limits _{k=i}^{i+2}\mathop {\sum }\limits _{l=j-1}^{j+1}\left(D^{|_{gr}}_{k,l}\right)^2+\varepsilon \right] \nonumber \\ \omega ^{\leftarrow } &= 1/\left[ \mathop {\sum }\limits _{k=i-1}^{i+1}\mathop {\sum }\limits _{l=j-2}^{j}\left(D^{-_{gr}}_{k,l}\right)^2+\varepsilon \right] \nonumber \\ \omega ^{\rightarrow } &= 1/\left[ \mathop {\sum }\limits _{=i-1}^{i+1}\mathop {\sum }\limits _{l=j}^{j+2}\left(D^{-_{gr}}_{k,l}\right)^2+\varepsilon \right] \nonumber \\ \omega &= \omega ^{\uparrow }+\omega ^{\downarrow }+\omega ^{\leftarrow }+\omega ^{\rightarrow } \end{aligned}$$

Here, all weights are normalized to the [0, 1] interval by dividing the sum $\omega$ for the sake of simplicity. In the subsequent section, all calculated weights are normalized using the same way. Except for the previous weight factor, supplementary information from main diagonal and anti-diagonal directions is used to have a better decision in a texture region. Because we add new four directional weights, it provides the feasibility of improving the green channel result by updating the initial color difference estimates. Another four directional weights are

8 $$\begin{aligned} \omega ^{\nwarrow } &= 1/\left[ \left( \mathop {\sum }\limits _{k=0}^{2}\mathop {\sum }\limits _{l=0}^{2}\left(D^{\backslash _{gr}}_{i-k,j+l-k-1}\right)^2\right) +\varepsilon \right] \nonumber \\ \omega ^{\searrow } &= 1/\left[ \left( \mathop {\sum }\limits _{k=0}^{2}\mathop {\sum }\limits _{l=0}^{2}\left(D^{\backslash _{gr}}_{i+k,j+l+k-1}\right)^2\right) +\varepsilon \right] \nonumber \\ \omega ^{\nearrow } &= 1/\left[ \left( \mathop {\sum }\limits _{k=0}^{2}\mathop {\sum }\limits _{l=0}^{2}\left(D^{/_{gr}}_{i-k,j+l+k-1}\right)^2\right) +\varepsilon \right] \nonumber \\ \omega ^{\swarrow } &= 1/\left[ \left( \mathop {\sum }\limits _{k=0}^{2}\mathop {\sum }\limits _{l=0}^{2}\left(D^{/_{gr}}_{i+k,j+l-k-1}\right)^2\right) +\varepsilon \right] \end{aligned}$$

In next part, we eventually reach a green-red color difference for estimating the missing green values.

9 $$\begin{aligned} GR_{i,j}=GR_{i,j}*(1-w_1)+{\mathbf{M}}_{1}\otimes {\mathbf{GR}}_{i-2:i+2,j-2:j+2} \end{aligned}$$

where

10 $$\begin{aligned} {\mathbf{M}}_{1}=\left[ \begin{array}{ccccc} (1-w_2)\times w_1\times \omega ^{\nwarrow }&\quad{}0&\quad{}w_2\times w_1\times \omega ^{\uparrow }&\quad{}0&\quad{}(1-w_2)\times w_1\times \omega ^{\nearrow }\\ 0&\quad{}0&\quad{}0&\quad{}0&\quad{}0\\ w_2\times w_1\times \omega ^{\leftarrow }&\quad{}0&\quad{}0&\quad{}0&\quad{}w_2\times w_1\times \omega ^{\rightarrow }\\ 0&\quad{}0&\quad{}0&\quad{}0&\quad{}0\\ (1-w_2)\times w_1\times \omega ^{\swarrow }&\quad{}0&\quad{}w_2\times w_1\times \omega ^{\downarrow }&\quad{}0&\quad{}(1-w_2)\times w_1\times \omega ^{\searrow }\\ \end{array}\right] \end{aligned}$$

In experiments, we set $w_1=0.6$ and $w_2=0.8$. In our method, two group of weights (one group is from the horizontal and vertical directions, another is from the diagonal direction) are build to be better suited for representing neighbor information. Ultimate estimation to the green value at the red pixel is designed by

11 $$\begin{aligned} G_{i,j}=R_{i,j}+GR_{i,j} \end{aligned}$$

For the green/blue row and column, the same procedures as above can be performed. Until this step, all the green pixels have been interpolated.

### Red/blue channel estimation at blue/red position

After the fulfillment of the green channel, we initially reconstruct the red and blue value at the blue and red corresponding pixel. Because the interpolations of red and blue channels are similar at this time, without loss of generality, we only discuss the red channel reconstruction. These red pixels are reconstructed based on $7\times 7$ windows. The similar weight matrix is also proposed in Pekkucuksen and Altunbasak (2013)

12 $$\begin{aligned} {\mathbf{M}}_2=\frac{1}{20}\times \left[ \begin{array}{ccccccc} 0&{}\quad 0&{}\quad -1&{}\quad 0&{}\quad -1&{}\quad 0&{}\quad 0\\ 0&{}\quad 0 &{}\quad 0&{}\quad 0&{}\quad 0&{}\quad 0&{}\quad 0\\ -1&{}\quad 0&{}\quad 7&{}\quad 0&{}\quad 7&{}\quad 0&{}\quad -1\\ 0&{}\quad 0&{}\quad 0&{}\quad 0&{}\quad 0&{}\quad 0&{}\quad 0\\ -1&{}\quad 0&{}\quad 7&{}\quad 0&{}\quad 7&{}\quad 0&{}\quad -1\\ 0&{}\quad 0&{}\quad 0&{}\quad 0&{}\quad 0&{}\quad 0&{}\quad 0\\ 0&{}\quad 0&{}\quad -1&{}\quad 0&{}\quad -1&{}\quad 0&{}\quad 0\\ \end{array}\right] \end{aligned}$$

The color difference between green and red is derived from the local window at the pixel coordinate (*i*, *j*). In essence, this matrix is similar to the negative Laplace filter in order to strengthen the center region.

13 $$\begin{aligned} R_{i,j}=G_{i,j}-{\mathbf{M}}_2\otimes {\mathbf{GR}}_{i-3:i+3,j-3:j+3} \end{aligned}$$

where ${\mathbf{GR}}_{i-3:i+3,j-3:j+3}$ is the G/R color difference and the operator $\otimes$ denotes element-wise multiplication in the equal size matrix and subsequent summation. Furthermore, each point can be refined using the Eq. (14).

14 $$\begin{aligned} R_{i,j}=G_{i,j}-{\mathbf{M}}_3\otimes {\mathbf{GR}}_{i-1:i+1,j-1:j+1} \end{aligned}$$

where

15 $$\begin{aligned} {\mathbf{M}}_3=\left[ \begin{array}{ccc} 0&{}0.5\omega ^{\uparrow }&{}0\\ 0.5\omega ^{\leftarrow }&{}0.5&{}0.5\omega ^{\rightarrow }\\ 0&{}0.5\omega ^{\downarrow }&{}0\\ \end{array}\right] \end{aligned}$$

### Red and blue channel at green component position

In the following, we interpolate the red and blue pixels at green components located in all even coordinates pixels. This procedure includes two phases. In the first phase, we estimate the red value in the green channel using the horizontal and vertical color difference. Points in the even row are interpolated. This procedure can avoid the sensitive estimation because the parameter from the single row and column is enlarged using the inverse ratio. Since the red/green color difference in the interlaced diagonal direction, in the second phase, we reconstruct red/green color difference using fully directional weight in the rest location (all odd coordinates pixels). The detailed interpolating equation is followed by the Eq. (16).

16 $$\begin{aligned} R_{i,j}=G_{i,j}-{\mathbf{M}}_4\otimes {\mathbf{GR}}_{i-1:i+1,j-1:j+1} \end{aligned}$$

where

17 $$\begin{aligned} {\mathbf{M}}_4=\left[ \begin{array}{ccc} 0&{}\quad \omega ^{\uparrow }&{}\quad 0\\ \omega ^{\leftarrow }&{}\quad 0&{}\quad \omega ^{\rightarrow }\\ 0&{}\quad \omega ^{\downarrow }&{}\quad 0\\ \end{array}\right] \end{aligned}$$

The interpolation is based on the prior value in the same color channel. After pixels in the even rows are interpolated, these recovered color value can serve as the interpolation processing in the odd rows. These prior results can further improve the performance. The Eq. (18) is re-performed at the corresponding. This refinement scheme elaborates the color difference based on the previous estimation.

18 $$\begin{aligned} R_{i,j}=G_{i,j}-{\mathbf{M}}_5\otimes {\mathbf{GR}}_{i-1:i+1,j-1:j+1} \end{aligned}$$

where

19 $$\begin{aligned} {\mathbf{M}}_5=\left[ \begin{array}{ccc} -0.25w_1&{}2w_2\times \omega ^{\uparrow }&{}-0.25w_1\\ 2w_2\times \omega ^{\leftarrow }&{}0&{}2w_2\times \omega ^{\rightarrow }\\ -0.25w_1&{}2w_2\times \omega ^{\downarrow }&{}-0.25w_1\\ \end{array}\right] \end{aligned}$$

**Fig. 2:**
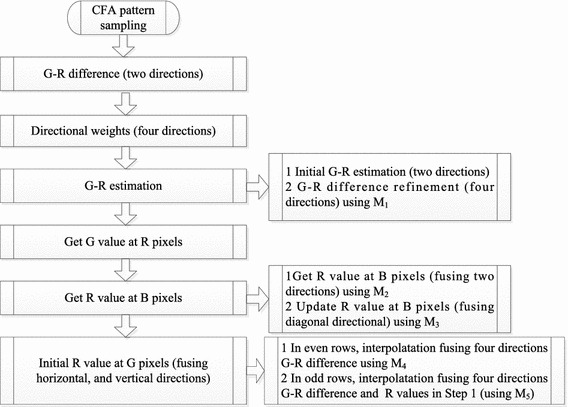
Demosaicking processing chain. Two directions mean horizontal and vertical directions, and four directions mean horizontal, vertical and two diagonal directions

The missing blue values at the green component positions are executed in the likelihood way. The whole demosaicking processing chain is shown in Fig. 2. We only give the processing between green and red channels. G-B estimation is same as this processing. The matrices $M_i, i=1,2,3,4,5$ corresponds to the processing chain in this flowchart.

## Experimental verification

**Fig. 3:**
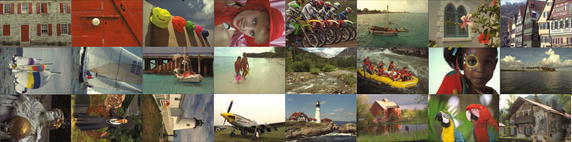
Testing images in Kodak dataset (Refers as image 1 to image 24 from *left-to-right* and *top-to-bottom*)

**Fig. 4:**
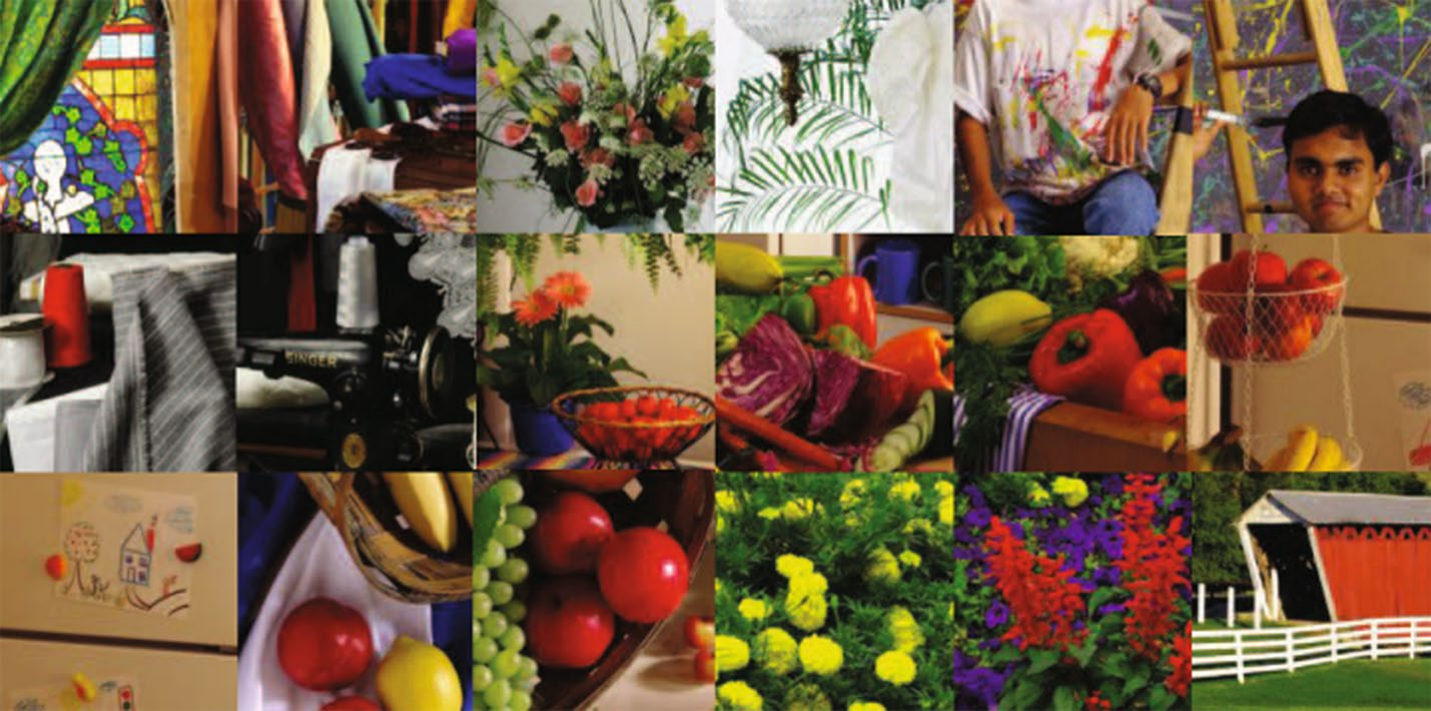
Testing images in McMaster dataset (Refers as image 1 to image 18 from *left-to-right* and *top-to-bottom*)

In this section, we investigate the performance of the proposed demosaicking method by analyzing two known benchmark image sets. One image source is the Kodak database containing 24 films captured and then digitized at the resolution of $512\times 768$. We number these images from one to twenty four. These images is illustrated in Fig. 3. Another image source is McMaster sets containing 18 color images with the size of $512\times 512$. The McMaster image set is shown in Fig. 4. We sample them according to the Bayer pattern to obtain a grey image and recreate them with different demosaicking techniques, comparing the interpolated images with the original ones. Menon and Calvagno (2011) have systematically investigated the performance beyond ten methods for the Kodak data sets tested in the previous works. Here, we select some representative algorithms and compare the performance of the proposed algorithms, including directional linear minimum mean square-error estimation (DL) (Zhang and Wu 2005), alternating projections (AP) (Gunturk et al. 2002), adaptive filtering (AF) (Lian et al. 2007), integrated gradients (IGD) (Chung and Chan 2010), regularization approaches to demosaicing (RAD) (Menon and Calvagno 2009) and the state-of-art multiscale gradients (MG) (Pekkucuksen and Altunbasak 2013) and residual interpolation (RI) (Kiku et al. 2016) algorithms. Note that we have implemented the MG method and found that it has a slightly performance difference compared to the results occurred at the reference Pekkucuksen and Altunbasak (2013) and the average PSNR values coincide exactly with the MG method. Since this implementation ignores the processing of pixels at the border, we exclude those pixels whose distance to the border is fewer than 10 pixels.

We evaluate these algorithms using objective quality metric color-peak signal-to-noise ratio (CPSNR) and structural similarity index (SSIM) value (Wang et al. 2004). CPSNR is calculated by $\text {CPSNR}=10log_{10}(255^2/\text {CMSE})$. $\text {CMSE}$ can be obtained by

20 $$\begin{aligned} \text {CMSE}=\frac{1}{3MN}\mathop {\sum }\limits _{i=r,g,b}\mathop {\sum }\limits _{x=1}^{M}\mathop {\sum }\limits _{y=1}^{N}(f(x,y,i)-f_d(x,y,i))^2 \end{aligned}$$

where *f* and $f_d$ represent the original and demosaicking image of size $M\times N$ each. The quantitative comparison (CPSNR) is summarized in Tables 1 and 2 for eight algorithms. The average CPSNR values of the proposed method are better than the closest method (MG) by 0.13 and 0.54 dB in Kodak and McMaster sets. The results of MG is directly quoted from their work Pekkucuksen and Altunbasak (2013) in the Kodak image set. For the McMaster dataset, the performance of RI is the best. The proposed method achieves the best performance in color difference series.

**Table 1:** CPSNR values for various algorithms in Kodak set

No.	RI	DL	AP	AF	IGD	RAD	MG	Prop.
1	36.32	38.52	37.82	37.56	40.09	37.41	39.87	39.58
2	40.47	40.93	39.65	40.70	41.06	40.02	41.77	41.82
3	42.57	42.75	41.61	42.68	43.42	42.34	43.72	44.06
4	41.17	41.09	40.03	41.05	41.08	41.27	41.13	42.04
5	37.29	38.10	37.54	38.03	38.43	38.01	39.05	39.42
6	38.74	40.27	38.61	38.03	41.15	39.03	41.38	41.28
7	42.57	42.39	41.74	42.90	42.70	42.78	43.51	44.01
8	34.49	36.08	35.30	35.22	37.46	34.98	37.56	37.39
9	41.92	42.86	41.84	42.56	43.38	41.96	43.96	43.95
10	42.04	42.61	42.06	42.69	42.95	42.42	43.20	43.54
11	38.84	40.09	39.24	39.33	40.81	39.22	41.36	41.29
12	42.93	43.53	42.65	42.77	44.25	43.10	44.45	44.72
13	32.52	34.81	34.37	33.76	36.14	33.82	36.00	35.72
14	37.43	37.03	35.82	37.15	37.33	36.36	37.97	38.54
15	39.14	39.87	39.37	39.83	39.92	40.15	40.30	40.65
16	42.45	43.83	41.82	41.14	44.61	42.36	44.86	44.86
17	40.70	41.86	41.41	41.38	41.99	41.17	42.32	42.45
18	36.04	37.45	37.36	37.16	37.74	36.93	38.22	38.26
19	39.49	40.90	39.87	40.00	41.73	39.38	42.17	41.97
20	40.28	41.27	40.68	41.11	41.76	40.69	42.16	42.21
21	37.70	39.17	38.92	38.67	40.14	38.50	40.31	40.14
22	38.23	38.46	37.84	38.50	38.63	38.21	39.05	39.20
23	43.08	43.30	41.87	43.14	43.33	42.71	44.02	44.32
24	34.43	35.52	34.68	34.84	35.36	35.09	35.69	35.70
Ave.	39.201	40.112	39.253	39.592	40.645	39.497	41.00	41.130

Comparing with PSNR which is an statistical average quality measure, SSIM value achieves high correlation with human perception of image quality, which is designed on the basis of characteristics of human visual system. For computing SSIM, we use the code provided by the original authors with default parameters and average three color channel values. Tables 3 and 4 show that average SSIM values of the proposed algorithm outperform other comparative methods.

**Table 2:** CPSNR values for various algorithms in McMaster set

No.	RI	DL	AP	AF	IGD	RAD	MG	Prop.
1	29.41	26.98	25.59	27.35	27.17	26.28	27.19	27.68
2	35.33	33.68	32.44	33.88	33.61	33.04	33.79	34.13
3	34.03	32.59	31.62	33.07	32.82	32.69	33.02	33.45
4	37.97	34.32	33.20	36.03	35.32	36.29	35.74	36.59
5	34.41	31.27	29.94	31.72	31.34	30.87	31.29	32.02
6	38.80	33.84	31.98	34.24	34.02	33.54	33.83	34.73
7	37.01	38.64	37.79	37.88	39.40	37.62	39.09	38.96
8	37.27	37.45	36.55	37.92	37.55	37.26	37.71	38.24
9	36.82	34.41	33.25	35.42	34.85	34.48	34.89	35.69
10	39.08	36.34	34.95	36.92	36.72	36.15	36.63	37.29
11	40.17	37.25	35.96	37.64	37.38	37.03	37.42	38.00
12	39.80	36.60	35.73	37.07	36.89	36.57	36.92	37.45
13	40.61	38.79	37.42	39.25	38.99	38.16	38.98	39.49
14	39.07	37.23	36.24	37.28	37.07	36.65	37.21	37.61
15	39.22	37.27	36.32	37.55	37.18	37.02	37.28	37.76
16	35.42	30.46	29.05	30.56	30.23	30.06	30.32	30.95
17	33.18	29.31	27.98	30.65	29.92	29.73	39.52	30.51
18	36.41	33.92	32.49	34.37	34.03	33.27	34.18	34.40
Ave.	36.890	34.463	33.249	34.933	34.693	34.260	34.723	35.276

**Table 3:** SSIM values for various algorithms in Kodak set

No.	RI	DL	AP	AF	IGD	RAD	MG	Prop.
1	0.9788	0.9873	0.9851	0.9844	0.9908	0.9836	0.9905	0.9900
2	0.9755	0.9776	0.9673	0.9763	0.9774	0.9719	0.9798	0.9804
3	0.9872	0.9883	0.9853	0.9877	0.9892	0.9871	0.9897	0.9901
4	0.9815	0.9825	0.9779	0.9823	0.9828	0.9825	0.9840	0.9853
5	0.9877	0.9889	0.9865	0.9890	0.9895	0.9885	0.9907	0.9914
6	0.9853	0.9889	0.9854	0.9842	0.9903	0.9864	0.9906	0.9907
7	0.9897	0.9899	0.9879	0.9904	0.9902	0.9897	0.9910	0.9916
8	0.9796	0.9864	0.9845	0.9840	0.9888	0.9838	0.9891	0.9889
9	0.9786	0.9854	0.9838	0.9848	0.9864	0.9836	0.9870	0.9871
10	0.9827	0.9866	0.9856	0.9866	0.9872	0.9855	0.9879	0.9880
11	0.9845	0.9876	0.9842	0.9852	0.9891	0.9851	0.9899	0.9900
12	0.9852	0.9878	0.9853	0.9860	0.9889	0.9864	0.9891	0.9895
13	0.9685	0.9817	0.9817	0.9786	0.9862	0.9786	0.9859	0.9853
14	0.9823	0.9846	0.9789	0.9835	0.9859	0.9811	0.9874	0.9880
15	0.9747	0.9787	0.9733	0.9786	0.9788	0.9793	0.9798	0.9810
16	0.9873	0.9903	0.9871	0.9858	0.9912	0.9879	0.9915	0.9916
17	0.9853	0.9886	0.9880	0.9880	0.9889	0.9875	0.9897	0.9897
18	0.9756	0.9810	0.9800	0.9809	0.9816	0.9802	0.9831	0.9835
19	0.9800	0.9858	0.9844	0.9839	0.9868	0.9835	0.9876	0.9875
20	0.9756	0.9778	0.9756	0.9774	0.9782	0.9760	0.9788	0.9791
21	0.9781	0.9831	0.9817	0.9821	0.9840	0.9810	0.9848	0.9849
22	0.9759	0.9767	0.9739	0.9773	0.9766	0.9767	0.9783	0.9792
23	0.9843	0.9851	0.9822	0.9854	0.9848	0.9841	0.9859	0.9866
24	0.9824	0.9861	0.9843	0.9855	0.9866	0.9847	0.9874	0.9877
Ave.	0.9811	0.9849	0.9821	0.9837	0.9858	0.9831	0.9866	0.9870

**Table 4:** SSIM values for various algorithms in McMaster set

No.	RI	DL	AP	AF	IGD	RAD	MG	Prop.
1	0.9236	0.8686	0.8289	0.8746	0.8716	0.8425	0.8707	0.8797
2	0.9451	0.9238	0.9014	0.9254	0.9208	0.9103	0.9212	0.9256
3	0.9718	0.9569	0.9458	0.9617	0.9580	0.9551	0.9581	0.9614
4	0.9896	0.9830	0.9774	0.9861	0.9847	0.9842	0.9854	0.9866
5	0.9577	0.9168	0.8913	0.9212	0.9183	0.9019	0.9162	0.9234
6	0.9690	0.9240	0.8937	0.9306	0.9272	0.9200	0.9236	0.9344
7	0.9680	0.9741	0.9700	0.9719	0.9764	0.9696	0.9754	0.9754
8	0.9724	0.9660	0.9585	0.9701	0.9681	0.9626	0.9660	0.9684
9	0.9611	0.9380	0.9184	0.9451	0.9402	0.9310	0.9396	0.9441
10	0.9711	0.9516	0.9362	0.9553	0.9532	0.9474	0.9516	0.9561
11	0.9728	0.9407	0.9262	0.9444	0.9419	0.9390	0.9408	0.9466
12	0.9642	0.9510	0.9422	0.9536	0.9509	0.9486	0.9505	0.9535
13	0.9531	0.9443	0.9322	0.9471	0.9429	0.9391	0.9423	0.9452
14	0.9566	0.9437	0.9318	0.9446	0.9413	0.9376	0.9412	0.9448
15	0.9561	0.9356	0.9207	0.9387	0.9328	0.9298	0.9327	0.9373
16	0.9659	0.8880	0.8666	0.8954	0.8915	0.8884	0.8882	0.9009
17	0.9505	0.8761	0.8273	0.9012	0.8835	0.8752	0.8748	0.8913
18	0.9670	0.9435	0.9288	0.9474	0.9430	0.9375	0.9434	0.9457
Ave.	0.9620	0.9348	0.9165	0.9397	0.9359	0.9289	0.9345	0.9400

**Fig. 5:**
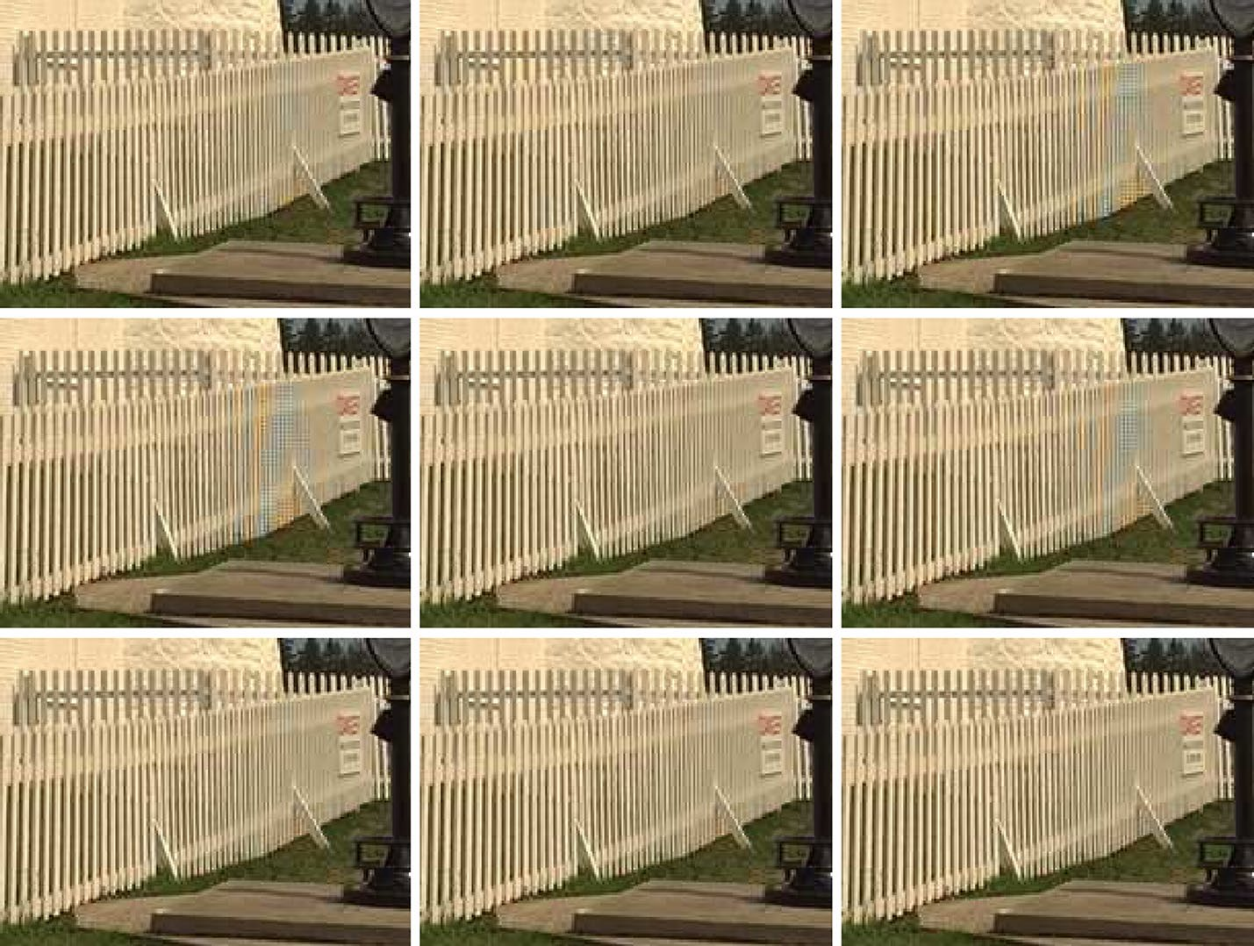
Local of image (No. 19) in the Kodak set, using different methods referred as image 1 to image 9 from *left-to-right* and *top-to-bottom*. (*1*) RI, (*2*) DL, (*3*) AP, (*4*) AF, (*5*) IGD, (*6*) RAD, (*7*) MG, (*8*) Proposed, (*9*) original image

It is shown in Fig. 5 that the visual quality comparison of local roof in image (No. 19) of the Kodak set is executed by various interpolation methods. We can see some obvious color artifact using other different methods. Demosaicking image of the proposed method is the most slightly blurred. On the whole, the proposed method produces the most desired visual quality.

Table 5 gives a comparison of computational complexity among the algorithms. The simulations have been conducted in the Matlab platform running on the desktop PC (Intel i7-2600 CPU). It is stated that the proposed is slower than MG, because the proposed method provides the improvement based on MG and keeps the most MG’s architecture. However, AF is the fastest among all the algorithms.

**Table 5:** Computation time (seconds) for various algorithms

Dataset	RI	DL	AP	AF	IGD	RAD	MG	Prop.
Kodak	2.72	18.3	1.5	0.3	–	0.4	8.2	13.6
McMaster	1.63	11.5	0.9	0.2	–	0.3	5.1	8.4

## Conclusion

In this paper, an efficiently fully directional estimation-based demosaicking method is developed. Computational weighting parameters adopted here inherit the actual result from eight directional information. Unlike the other standard weight allocation algorithms, new approach allows the adaptive adjustment satisfied to local interpolation and optimal target. The proposed method need integrate the weight allocation interpolation, and finally perform an entirely demosaicking application. At the same time, the quality of the resulting images produced by the proposed approach is better in perception than that produced by those without priority estimation. Experimental results show that the proposed method is more efficient than other methods such as DL, AP, AF, IGD, RAD as well as the state-of-art MG and RI algorithms. The results of PSNR and SSIM proves that the proposed method is valid, and can obtain high performance accuracy and good results in the application.

## References

[CR1] Adams JE, Hamilton JF, Jr (1996) Adaptive color plane interpolation in single color electronic camera. U.S. Patent 5506619

[CR2] Bayer B-E (1976) Color imaging array. U.S. Patent 3971065

[CR3] Chung K-H, Chan Y-H (2010). A low complexity color demosaicing algorithm based on integrated gradient. J Electron Imag.

[CR4] Gunturk BK, Altunbasak Y, Mersereau RM (2002). Color plane interpolation using alternating projections. IEEE Trans Image Process.

[CR5] Jaiswal S, Au O-C, Jakhetiya V, Yuan Y, Yang H (2014) Exploitation of inter-color correlation for color image demosaicking. In: Proceedings on IEEE international conference on image processing, pp. 1812–1816

[CR8] Kiku D, Monno Y, Tanaka M, Okutomi M (2013) Residual interpolation for color image demosaicking. In: Proceedings on IEEE international conference on image processing, pp. 2304–2308

[CR6] Kiku D, Monno Y, Tanaka M, Okutomi M (2014). Minimized-Laplacian residual interpolation for color image demosaicking. Proc IS&T/SPIE Electron Imaging.

[CR7] Kiku D, Monno Y, Tanaka M, Okutomi M (2016). Beyond color difference: residual interpolation for color image demosaicking. IEEE Trans Image Process.

[CR9] Kim J, Jeon G, Jeong J (2014). Demosaicking using geometric duality and dilated directional differentiation. Opt Commun.

[CR10] Li X, Gunturk B, Zhang L (2008). Image demosaicking: a systematic survey. Proc SPIE Vis Commun Image Process.

[CR11] Lian N-X, Chang LL, Tan Y-P, Zagorodnov V (2007). Adaptive filtering for color filter array demosaicking. IEEE Trans Image Process.

[CR12] Menon D, Calvagno G (2009). Regularization approaches to demosaicking. IEEE Trans Image Process.

[CR13] Menon D, Calvagno G (2011). Color image demosaicking: an overview. Signal Process Image Commun.

[CR14] Monno Y, Kiku D, Tanaka M, Okutomi M (2015) Adaptive residual interpolation for color image demosaicking. In: Proceedings on IEEE international conference on image processing, pp. 3861–3865

[CR15] Pekkucuksen I, Altunbasak Y (2013). Multiscale gradients-based color filter array interpolation. IEEE Trans Image Process.

[CR16] Wang Z, Bovik AC, Sheikh HR, Simoncelli EP (2004). Image quality assessment: from error visibility to structural similarity. IEEE Trans Image Process.

[CR17] Ye W, Ma K-K (2015). Color image demosaicing using iterative residual interpolation. IEEE Trans Image Process.

[CR19] Zhang L, Wu XL (2005). Color demosaicking via directional linear minimum mean square-error estimation. IEEE Trans Image Process.

[CR18] Zhang F, Wu X, Yang X, Zhang W, Zhang L (2009). Robust color demosaicking with adaptation to varying spectral correlations. IEEE Trans Image Process.

[CR20] Zhou D, Shen X, Dong W (2012). Colour demosaicking with directional filtering and weighting. IET Image Process.

